# New Statistical Models for Copolymerization

**DOI:** 10.3390/polym8060240

**Published:** 2016-06-22

**Authors:** Martin S. Engler, Kerstin Scheubert, Ulrich S. Schubert, Sebastian Böcker

**Affiliations:** 1 Chair of Bioinformatics, Friedrich Schiller University Jena, Ernst-Abbe-Platz 2, 07743 Jena, Germany; martin.engler@uni-jena.de (M.S.E.); kerstin.scheubert@uni-jena.de (K.S.); 2 Laboratory of Organic and Macromolecular Chemistry (IOMC), Friedrich Schiller University Jena, Humboldtstr. 10, 07743 Jena, Germany; ulrich.schubert@uni-jena.de; 3 Jena Center for Soft Matter (JCMS), Friedrich Schiller University Jena, Philosophenweg 7, 07743 Jena, Germany

**Keywords:** copolymer kinetics, copolymer fingerprint, Markov model

## Abstract

For many years, copolymerization has been studied using mathematical and statistical models. Here, we present new Markov chain models for copolymerization kinetics: the Bernoulli and Geometric models. They model copolymer synthesis as a random process and are based on a basic reaction scheme. In contrast to previous Markov chain approaches to copolymerization, both models take variable chain lengths and time-dependent monomer probabilities into account and allow for computing sequence likelihoods and copolymer fingerprints. Fingerprints can be computed from copolymer mass spectra, potentially allowing us to estimate the model parameters from measured fingerprints. We compare both models against Monte Carlo simulations. We find that computing the models is fast and memory efficient.

## 1. Introduction

Copolymerization is a random process, where two or more monomer species are mixed to form polymer chains. We investigate binary copolymers with two monomer types A and B. In the past, several approaches to model copolymerization were proposed. The well-known terminal model by Mayo and Lewis describes four propagation reactions and is determined by the reactivity ratios of the monomers [1]. There are three different computational approaches to such a basic reaction scheme and each approach has certain disadvantages. The reaction scheme can be modeled as a set of ordinary differential equations (ODE), a discrete Markov chain or simulated with Monte Carlo methods.

Mayo and Lewis described the scheme as a set of ODEs and deduced the copolymer equation, which provides the copolymer composition. This set of ODEs can be solved fast but does not convey any information on the chain sequences. Kryven and Iedema advanced the ODE approach by applying population balance equations [2]. They showed the importance of recovering “distributions in a full form rather than averages, since average values may often be far from the most frequently occurring ones” ([2], p. 305). They were able to extract simple sequence patterns, but not the full distribution of sequences.

Markov and Hidden Markov models are frequently used in the analysis of polymers and biopolymers, for example de novo peptide sequencing [3], detection of gene promoter regions [4], or prediction of quantitative structure–property relationships [5,6,7,8] for cellular recognition [9], or drug–DNA [10] or protein–protein interactions [11]. The transformation of the traditional Mayo–Lewis model to a Markov chain is straightforward and the resulting Markov chain can be used to compute the probability of a single copolymer chain [12], but not the distribution of all chains.

Gillespie introduced Monte Carlo methods to cheminformatics to simulate chemical reactions [13]. Gillespie’s algorithm has been frequently used to simulate copolymerizations by randomly growing copolymer chains [14,15,16]. Several times, Monte Carlo simulations have been evaluated against experimental data [17,18,19] and it has been shown that Gillespie’s algorithm can be used to compute copolymer fingerprints [20,21,22]. However, Monte Carlo simulations are time- and memory-intensive, in particular if an accurate representation of the distribution of copolymer chains is desired.

The distribution of copolymer chains can be represented using fingerprints. A copolymer fingerprint is the 2D compositional distribution, i.e., the abundance of each possible combination of monomer counts. The fingerprints can be estimated experimentally using mass spectrometry (MS), though the computational transformation of the experimental data to a fingerprint is a non-trivial problem [23]. Matrix-assisted laser desorption/ionization time-of-flight (MALDI-TOF) MS is frequently applied to characterize (co-)polymers [24,25] and can be used to estimate fingerprints [23,26,27,28]. Most recently, we proposed a method to correct fingerprints for mass discrimination effects [29] a long-known issue of MALDI ionization [30,31,32,33].

In this publication, we propose two new Markov chain models for copolymerization kinetics: the Bernoulli and the Geometric model based on a simple reaction scheme. Different to Mayo and Lewis [1], our model allows for variable chain lengths and time-dependent monomer probabilities. The accuracy of Monte Carlo simulations depends on the number of simulated chains, the simulated distribution converges to the true distribution with an increasing number. This makes accurate computations time- and memory-intensive. In contrast to Monte Carlo simulations, our models are exact and fast. We implement a simple copolymerization scheme using ODEs and Monte Carlo simulations. We verify the Monte Carlo simulations with the ODE system. We evaluate our models against the fingerprints and copolymer chains computed by Monte Carlo.

## 2. Materials and Methods

For evaluation against Monte Carlo methods, we consider the following simple copolymerization scheme. We denote an active center as X${}^{•}$, and a polymer chain ending with X as ~X, where X can be one of the monomers A or B, or initiator I. We model two types of reactions, initiation and propagation reactions:

$$\begin{array}{c} \begin{array}{c} I+A\overset{k_{IA}}{⟶}\;∼A^{•}\\ I+B\overset{k_{IB}}{⟶}\;∼B^{•} \end{array}\}\text{Initiation}\\ \begin{array}{c} ∼A^{•}+A\overset{k_{AA}}{⟶}\;∼A^{•}\\ ∼A^{•}+B\overset{k_{AB}}{⟶}\;∼B^{•}\\ ∼B^{•}+A\overset{k_{BA}}{⟶}\;∼A^{•}\\ ∼B^{•}+B\overset{k_{BB}}{⟶}\;∼B^{•} \end{array}\}\text{Propagation}. \end{array}$$

We use the reaction rate coefficient $k_{AA}=1.0\;L\cdot {\mathrm{mol}}^{-1}\cdot s^{-1}$ and the reactivity ratios $r_{A}=r_{B}^{-1}$ with the values 0.01, 0.05, and 0.1, plus values in the range from 0.25 to 2.0 with step size 0.25. All other reaction rate coefficients are $1.0\;L\cdot {\mathrm{mol}}^{-1}\cdot s^{-1}$. The initial amounts of A, B, and I are $n_{A}=1\;\mathrm{mol}$, $n_{B}=2\;\mathrm{mol}$, and $n_{I}=0.0094\;\mathrm{mol}$, respectively. The reaction scheme was implemented as an ODE system and solved in Python.

For the Monte Carlo simulations, we use $10^{2}$ to $10^{6}$ polymer chains and the same parameters as for the ODE system. The simulations are stopped at full conversion of A and B or if the simulated reaction time reaches $10^{3}$ s. We repeat the simulations ten times for each reactivity ratio and chain number. We implemented the Monte Carlo simulation software in Java using the conventional Gillespie’s algorithm [13].

We compute the Monte Carlo fingerprints by calculating a histogram from the simulated polymer chains. To compare two fingerprints, we use the normalized root mean square error (NRMSE). The $NRMSE(M_{1},M_{2})$ between two matrices $M_{1}$ and $M_{2}$ of size n×m is defined as:

(1) $$NRMSE\left(M_{1},M_{2}\right)=100\times \frac{\sqrt{\frac{1}{n\times m}\left|\right|M_{1}-M_{2}{\left|\right|}_{2}^{2}}}{\max\left(M_{2}\right)}.$$

Monte Carlo simulations produce a large random sample of polymer chains *S*. If a model *M* can compute the likelihood of a single chain P(S|M), we can compare different models by computing and comparing the log likelihoods of the whole dataset *D*:

(2) $$P\left(D|M\right)=\sum_{S\in D}\log P\left(S|M\right).$$

We use this log likelihood to evaluate our models.

## 3. Results and Discussion

### 3.1. Bernoulli Model

#### 3.1.1. Chain Lengths

Consider the synthesis of a single polymer chain. We divide the continuous reaction time into *T* discrete time steps, which we call synthesis steps. At each step, there are two mutually exclusive events: adding a monomer or not. This random process is equivalent to conducting a series of *T* Bernoulli trials for every polymer chain and recording the chain lengths, i.e., how many monomers were added. Thus, the chain lengths are binomially distributed with parameters *T*, the number of trials, and $p_{M}$, the probability of adding a monomer.

#### 3.1.2. Fingerprint Model

We extend the model to describe copolymer fingerprints. At each of the *T* discrete synthesis steps, three mutually exclusive events are possible: adding monomer A, monomer B, or nothing. However, in general, the proportion of A to B changes during the synthesis; therefore, the probabilities of adding A or B change. We define the monomer probability parameters $p_{A}\left(t\right)$ and $p_{B}\left(t\right)$, with $p_{A}\left(t\right)+p_{B}\left(t\right)=1$ for all 1≤t≤T. $p_{A}$ and $p_{B}$ are vectors of length *T*, describing the probability of encountering a monomer A or B at each synthesis step.

We model copolymerization as an inhomogeneous Markov chain and call this basic model the *Bernoulli model*. We describe a copolymer fingerprint as a matrix *M* of size n×m, in which entry $M_{a,b}$ gives the relative abundance of a copolymer with *a* monomers of type A and *b* monomers of type B. The states of the Markov chain correspond to the fingerprint entries. The transition probabilities correspond to the three possible events: append A, B, or nothing. The transition probability from state $M_{a,b}$ to $M_{a+1,b}$ is the probability of adding a monomer $p_{M}$ times the probability of encountering an A at synthesis step *t*:

(3) $$P\left(M_{a,b}\to M_{a+1,b}\right)=p_{M}\times p_{A}\left(t\right).$$

Analogously, the transition probability from $M_{a,b}$ to $M_{a,b+1}$ is the probability of adding a monomer times the probability of encountering a B:

(4) $$P\left(M_{a,b}\to M_{a,b+1}\right)=p_{M}\times p_{B}\left(t\right).$$

The transition probability for staying in state $M_{a,b}$ is the probability of adding nothing:

(5) $$P\left(M_{a,b}\to M_{a,b}\right)=1-p_{M}.$$

All other transition probabilities are zero.

The starting distribution M(0) is a matrix of zeros, except for $M_{0,0}\left(0\right)=1$. This means that, before starting the synthesis, all chains have zero monomer repeating units A and B. To conform to standard Markov chain notation, let *M* be a row vector. Let *P* be the matrix of transition probabilities. Starting with M(0), the copolymer fingerprint at synthesis step *t* is:

(6) M(t)=M(t-1)×P(t).

We are interested in the fingerprint after the completed synthesis, which is the fingerprint at the last synthesis step M(T). The transition matrix *P* is sparse; thus, Equation (6) can be simplified for a>0 and b>0 to:

(7) $$\begin{array}{ccc} M_{a,b}\left(t\right) & = & p_{M}\times p_{A}\left(t\right)\times M_{a-1,b}\left(t-1\right)\\ & + & p_{M}\times p_{B}\left(t\right)\times M_{a,b-1}\left(t-1\right)\\ & + & \left(1-p_{M}\right)\times M_{a,b}\left(t-1\right). \end{array}$$

If a=0 or b=0, one needs to delete from Equation (7) the first or second term, respectively. In each synthesis step 1≤t≤T, we compute n×m fingerprint entries in constant time for each entry. Because n≤T and m≤T, the worst case running time is $O(T^{3})$. It is not necessary to save the fingerprints for each synthesis step as M(t) only depends on M(t-1); therefore, the memory requirement is $O(T^{2})$.

#### 3.1.3. Reactivity Ratios

Thus far, our model has not taken reactivity ratios into account. The probability of a reaction equals the probability of adding a certain monomer times the probability of encountering that monomer. However, the reactivity ratios are known to influence the copolymerization process. For example, if monomer A has a strong affinity for monomer B, a weak affinity for A, and monomer B has the reverse affinity, then the result will be an alternating copolymer. To this end, we define a new model: the *Bernoulli model with reactivity parameters*.

We define the reactivity parameters $p_{AA}$, $p_{AB}$, $p_{BA}$, and $p_{BB}$, which describe the probabilities of the reactions between the four possible pairings of chain ends and monomers. To be able to distinguish between chains ends, we use two fingerprints: $M^{A}$, the distribution of chains ending with A, and $M^{B}$, the distribution of chains ending with B. We are interested in the fingerprint after the final synthesis step *T*. The final fingerprint can be calculated by adding the final distributions of chains ending with A and B:

(8) $$M\left(T\right)=M^{A}\left(T\right)+M^{B}\left(T\right).$$

We define the transition probabilities for the four possible reactions of chain ends and monomers. For X∈{A,B}, the transition probabilities for adding A are:

(9) $$P\left(M_{a,b}^{X}\to M_{a+1,b}^{A}\right)=p_{M}\times c_{X}\times p_{XA}\times p_{A}\left(t\right).$$

Analogously, the transition probabilities for adding B are:

(10) $$P\left(M_{a,b}^{X}\to M_{a,b+1}^{B}\right)=p_{M}\times c_{X}\times p_{XB}\times p_{B}\left(t\right).$$

An important property of Markov chains is that the rows of the transition matrix sum to one. Introducing the reactivity parameters violated this property; therefore, we use the normalization coefficients $c_{A}$ and $c_{B}$ in the Equations (9) and (10). The normalization coefficients are defined as:

(11) $$c_{X}=\frac{1}{p_{XA}\times p_{A}\left(t\right)+p_{XB}\times p_{B}\left(t\right)}.$$

Because empty chains end neither with A nor with B, we define the initiator state I. The transition probabilities to start a chain are:

(12) $$\begin{array}{ccc} P(I\to M_{1,0}^{A}) & = & p_{M}\times p_{A}\left(t\right),\\ P(I\to M_{0,1}^{B}) & = & p_{M}\times p_{B}\left(t\right). \end{array}$$

The transition probabilities of the non-state-changing transitions are not affected by the reactivity parameters and are analogous to Equation (5). All other transition probabilities are zero. By applying the transition probabilities (Equations (9) and (10)) and the normalization coefficients (Equation (11)), the fingerprint $M^{A}$ can be calculated for a>0 and b>0 by:

(13) $$\begin{array}{ccc} M_{a,b}^{A}\left(t\right) & = & p_{M}c_{A}p_{AA}p_{A}\left(t\right)\times M_{a-1,b}^{A}\left(t-1\right)\\ & + & p_{M}c_{B}p_{BA}p_{A}\left(t\right)\times M_{a-1,b}^{B}\left(t-1\right)\\ & + & \left(1-p_{M}\right)\times M_{a,b}^{A}\left(t-1\right). \end{array}$$

Analogously, fingerprint $M^{B}$ is:

(14) $$\begin{array}{ccc} M_{a,b}^{B}\left(t\right) & = & p_{M}c_{A}p_{AB}p_{B}\left(t\right)\times M_{a,b-1}^{A}\left(t-1\right)\\ & + & p_{M}c_{B}p_{BB}p_{B}\left(t\right)\times M_{a,b-1}^{B}\left(t-1\right)\\ & + & \left(1-p_{M}\right)\times M_{a,b}^{B}\left(t-1\right). \end{array}$$

If a=0 or b=0, the appropriate terms can be deleted from Equations (13) and (14). For a=1,b=0 or b=1,a=0 Equations (13) and (14) change according to Equation (12).

The running time and memory requirements change by a constant factor; therefore, the worst case running time is still $O(T^{3})$ and memory is $O(T^{2})$.

### 3.2. Geometric Model

#### 3.2.1. Chain Length

The Bernoulli model that we introduced above used *T* discrete synthesis steps to add monomers: A, B or nothing. Adding a monomer or not is a Bernoulli trial and the resulting chain lengths are binomially distributed. However, in practice, polymer lengths often show a long-tailed distribution, which is usually modeled by a gamma distribution [12,34,35]. Here, we modify our discrete model for a long-tailed chain length distribution. The discrete equivalent to the continuous gamma distribution is the negative binomial distribution. A random variable following a negative binomial distribution with parameters *T* and *p* equals the sum of *T* independent geometrically distributed random variables with parameter 1-p. To this end, we model the discrete steps using the geometric distribution.

Consider the synthesis of a single polymer chain. In each synthesis step, the number of monomers, which are added to the chain, is random. The probability of adding *k* monomers follows a geometric distribution with parameter $p_{\epsilon }$, the “stop” probability:

(15) $$P_{G}\left(k\right)={\left(1-p_{\epsilon }\right)}^{k}p_{\epsilon }.$$

We call this the *Geometric model*.

#### 3.2.2. Fingerprint Model

Due the geometrically distributed number of monomers to add in each synthesis step, the number of possible transitions increases compared to the Bernoulli model. Given i≥0 and j≥0, the transition probability from $M_{a,b}$ to any state with equal or higher numbers of A and B is the number of combinations with *i* monomers of type A and *j* monomers of type B times the probability of adding i+j monomers times the probabilities of encountering *i* monomers of type A and *j* monomers of type B:

(16) P(Ma,b→Ma+i,b+j)=i+jj×PG(i+j)×pA(t)i×pB(t)j.

To save computation time, the number of combinations i+jj can be calculated using Pascal’s triangle. As with the Bernoulli model, the memory requirements are $O(T^{2})$. However, the running time increases to $O(T^{5})$ because we need to iterate over all possible *i* and *j*.

#### 3.2.3. Reactivity Ratios

Analogous to the Bernoulli model, we define a *Geometric model with reacitivity parameters*. We use the reactivity parameters $p_{AA}$, $p_{AB}$, $p_{BA}$, and $p_{BB}$ to model the reactivity ratios, the initiator state I, and two fingerprints $M^{A}$ and $M^{B}$ to describe the distributions of chains ending with A or B, respectively.

In contrast to the Bernoulli model, the Geometric model is able to add more than one monomer per synthesis step. We need to determine the reactivity parameters for all possible combinations of added A and B. Consider one synthesis step of the Markov chain: we say that we start in a state X∈{I,A,B}, if the last added monomer of all previous steps was nothing, A, or B, respectively. We stop in state Y∈{A,B}, if the last added monomer of this or any previous step is an A or B, respectively. To this end, we introduce the matrix $R^{XY}$. $R_{a,b}^{XY}$ is the probability of starting in state X, adding *a* monomers A, *b* monomers B, and ending in state Y. We define $R^{XY}$ as:

(17) $$\begin{array}{ccc} R_{a,b}^{XA} & = & R_{a-1,b}^{XA}\times p_{AA}+R_{a-1,b}^{XB}\times p_{BA},\\ R_{a,b}^{XB} & = & R_{a,b-1}^{XA}\times p_{AB}+R_{a,b-1}^{XB}\times p_{BB}. \end{array}$$

To compute $R^{XY}$ for each possible combination of X and Y, we need to know the initial values. If no monomer is added, we start and end in the same state:

(18) $$\begin{array}{cccc} R_{0,0}^{XX} & = & 1,\\ R_{0,0}^{XY} & = & 0 & \;\text{for}\;X\neq Y. \end{array}$$

If we start in the initiator state I and add one monomer, it is independent of the reactivity parameters:

(19) $$\begin{array}{ccc} R_{1,0}^{IA} & = & 1,\\ R_{0,1}^{IB} & = & 1. \end{array}$$

Analogous to the Bernoulli model with reactivity parameters, the rows of the transition matrix need to sum to one. We therefore define normalization coefficients and normalize the transition probabilities for all transitions that add the same number of monomers:

(20) $$c_{X}\left(k\right)=\frac{1}{\sum_{a+b=k}\left(R_{a,b}^{XA}+R_{a,b}^{XB}\right)\times p_{A}{\left(t\right)}^{a}\times p_{B}{\left(t\right)}^{b}}.$$

We now combine Equation (17) to Equation (20) to specify the transition probabilities for the Geometric model. For X∈{A,B}:

(21) $$P\left(M_{a,b}^{X}\to M_{a+i,b+j}^{Y}\right)=c_{X}\left(i+j\right)\times R_{i,j}^{XY}\times P_{G}\left(i+j\right)\times p_{A}{\left(t\right)}^{i}\times p_{B}{\left(t\right)}^{j}.$$

The transition probabilities from the initiator state I to any other state are given by:

(22) $$P\left(I\to M_{i,j}^{Y}\right)=c_{I}\left(i+j\right)\times R_{i,j}^{IY}\times P_{G}\left(i+j\right)\times p_{A}{\left(t\right)}^{i}\times p_{B}{\left(t\right)}^{j}.$$

The transition probability to not start a chain and stay in state I is:

(23) $$P\left(I\to I\right)=P_{G}\left(0\right).$$

We are interested in the fingerprint after the final synthesis step *T*. Analogous to the Bernoulli model, the final fingerprint can be calculated by adding the final distributions $M^{A}\left(T\right)$ and $M^{B}\left(t\right)$. Compared to the Geometric model without reactivity parameters, the running time and memory requirements change by a constant factor; therefore, the worst case running time is still $O(T^{5})$ and memory is $O(T^{2})$.

### 3.3. Single Chain Models

The Bernoulli and Geometric models described above compute the copolymer fingerprints, and the distribution of all chains over the numbers of monomer repeating units. However, an additional interesting question is: what is the likelihood of a single copolymer chain under a given model?

To compute the likelihood of a single chain, we only consider transitions which may lead to the chain in question and transitions which do not add a monomer, i.e., non-state-changing transitions. All other transition probabilities are zero. After progressing *T* synthesis steps, the likelihood of the chain is the probability of the last reachable state.

For example, let us compute the likelihood of the chain “ABB”. In addition to the non-state-changing transitions, the Bernoulli model would allow $M_{0,0}\to M_{1,0}$, $M_{1,0}\to M_{1,1}$, and $M_{1,1}\to M_{1,2}$. The likelihood of “ABB” is the probability of the state $M_{1,2}$. The likelihood under the Bernoulli model with reactivity parameters and the Geometric models can be computed analogously.

### 3.4. Parameter Estimation

The Bernoulli and Geometric models fully characterize the distribution of copolymer chains. Unfortunately, the true underlying distribution of copolymer chains is unknown and the Monte Carlo simulated chains are random samples. However, the larger the sample size is, the closer the empirical distribution is to the true distribution and the better we can use the sample to the evaluate our models.

**Figure 1:**
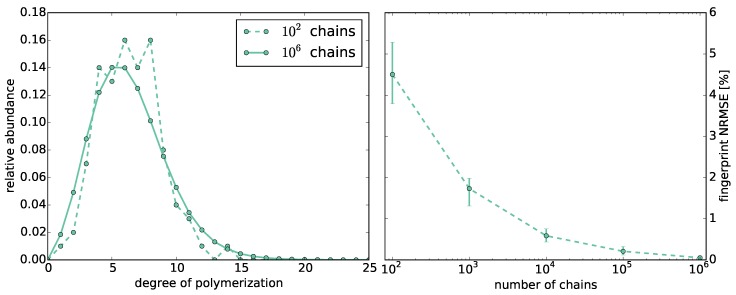
**Left**: Comparison of the distribution of chain lengths computed by the Monte Carlo simulations with $10^{2}$ *vs.* $10^{6}$ chains at reactivity ratio $r_{A}=1.0$; **Right**: Normalized root mean square error (NRSME) of the fingerprints computed by Monte Carlo simulations with different numbers of chains compared to the fingerprint computed from all chains produced by all Monte Carlo simulations at reactivity ratio $r_{A}=1.0$.

The accuracy of the Monte Carlo simulation strongly depends on the number of simulated chains (Figure 1, left). For $r_{A}=r_{B}^{-1}=1.0$, we compute 10 fingerprints *M* with $10^{2}$ to $10^{6}$ chains and compare them to the fingerprint $M_{total}$, which we compute using all $10\times \sum_{i=2}^{6}10^{i}=11,111,000$ chains (Figure 1, right). For comparison, we use the normalized root mean square error $NRMSE(M,M_{total})$.

The error decays with the number of chains. The lowest mean errors are ∼2% and ∼0.5% using $10^{5}$ and $10^{6}$ chains, respectively. We observe that the error for $10^{5}$ is still significantly above zero. Thus, if not stated otherwise, we use $10^{6}$ chains for the Monte Carlo simulations in the following.

For completeness, we evaluate the Monte Carlo simulations by comparing the simulated concentrations to the concentrations computed by solving the ordinary differential equation model of the living copolymerization (Supplementary Figure S1). The concentration curves are identical to the eye, strongly supporting the validity of the Monte Carlo simulations.

We now compare the Bernoulli and Geometric models to the Monte Carlo simulations. The reactivity parameters can be calculated from the reactivity ratios. For X,Y∈{A,B}, the reactivity parameters are:

(24) $$p_{XY}=\frac{r_{XY}}{r_{XA}+r_{XB}}.$$

Unfortunately, the other model parameters cannot be calculated intuitively from the Monte Carlo simulation parameters. In principle, it is possible to estimate the parameters by fitting the model fingerprint to the Monte Carlo fingerprint. However, to minimize the influence of the fitting algorithms, we apply a two-step estimation process.

First, we estimate the number of synthesis steps and the probability of adding monomers. According to the Bernoulli and Geometric model, the chain lengths follow a binomial or negative binomial distribution, respectively.

**Figure 2:**
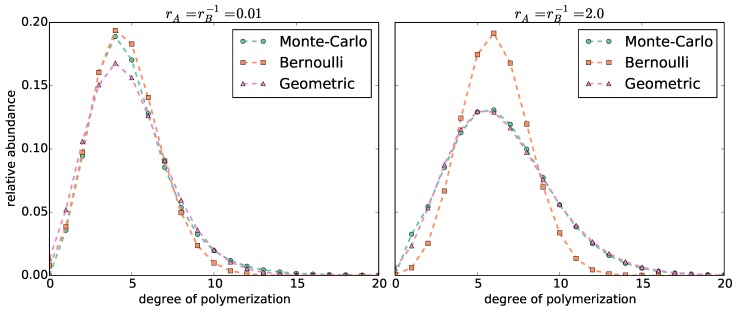
Comparison of the distribution of chain lengths computed by the Monte Carlo simulations with $r_{A}=0.01$ (**left**) and $r_{A}=2.0$ (**right**) *vs.* the length distributions computed by the Bernoulli and Geometric models.

We fit a binomial and a negative binomial probability mass function (pmf) to each copolymer length distribution (Figure 2 and Supplementary Figure S2). The length distributions become broader with increasing reactivity ratios $r_{A}=r_{B}-1$. The broader the distribution, the better it is approximated by a negative binomial pmf, and the worse by a binomial pmf. However, for narrow distributions ($r_{A}=r_{B}-1\leq 0.1$, which correspond to a standard deviation σ≤2.8), we do not observe such a clear distinction: The mode of the distribution is better approximated by the binomial pmf. In contrast, the negative binomial pmf is better able to fit the long tail of the distribution.

**Figure 3:**
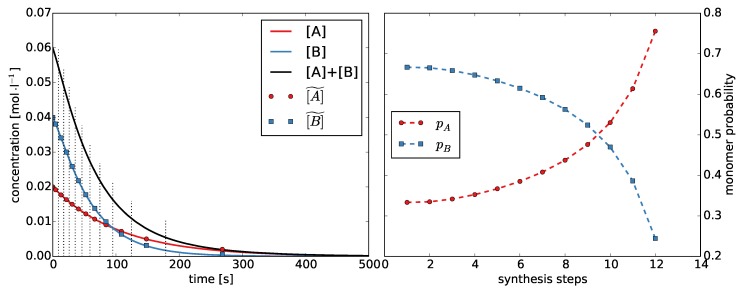
**Left**: Concentration of monomers $\left[A\right]$ and $\left[B\right]$ during the Monte Carlo simulation with $r_{A}=2.0$. We divided the time into discrete synthesis steps and determined the average concentrations $\tilde{\left[A\right]}$ and $\tilde{\left[B\right]}$; **Right**: Monomer probabilities $p_{A}$ and $p_{B}$ for each synthesis step calculated from the average concentrations.

Second, we estimate the monomer probabilities $p_{A}$ and $p_{B}$. Because we defined $p_{A}+p_{B}=1$, estimating $p_{A}$ is sufficient. We divide the reaction time of the Monte Carlo simulation into intervals. The number of intervals equals the number of synthesis steps *T*. We choose the left and right interval limits, such that the change in concentration is the same for each interval (Figure 3 and Supplementary Figure S3). We calculate the mean concentrations $\tilde{\left[A\right]}\left(t\right)$ and $\tilde{\left[B\right]}\left(t\right)$ for each interval 1≤t≤T. Then, the monomer probabilities $p_{A}\left(t\right)$ can be calculated as:

(25) $$p_{A}\left(t\right)=\frac{\tilde{\left[A\right]}\left(t\right)}{\tilde{\left[A\right]}\left(t\right)+\tilde{\left[B\right]}\left(t\right)}.$$

Please note that the parameter estimation using the concentrations from Monte Carlo simulations is for evaluation purposes only. When applying the models to experimental data, the parameters can be estimated by fitting the computed fingerprint to the observed fingerprint.

### 3.5. Model Evaluation

**Figure 4:**
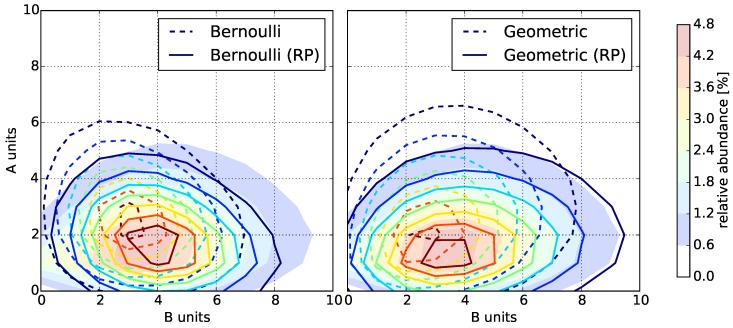
Copolymer fingerprint computed by the Monte Carlo simulation with $r_{A}=2.0$ (filled contours) compared to the fingerprints computed by the statistical models (solid and dashed contours). **Left**: Bernoulli model with and without reactivity parameters (RP); **Right**: Geometric model with and without reactivity parameters (RP).

**Figure 5:**
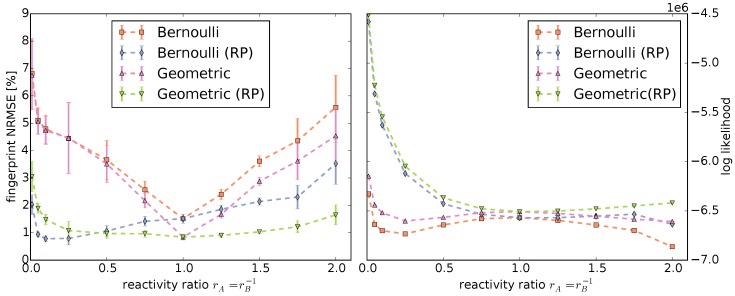
**Left**: Normalized root mean square error (NRMSE) of the copolymer fingerprints computed by Monte Carlo simulations compared to the fingerprints computed by the statistical models; **Right**: Log likelihoods of the polymer chains produced by the Monte Carlo simulations under the Bernoulli and Geometric models with and without RP. Note that the minimal and maximal log likelihoods are so close to the means that the error bars are indiscernible.

Determining the model parameters allows us to compare the fingerprints computed by our models to the Monte Carlo fingerprints (Figure 4 and Supplementary Figure S4). Additionally, we can compute the NRMSE of the Monte Carlo fingerprints vs. the model fingerprints (Figure 5, left).

Evidently, the reactivity parameters are crucial to model copolymerization. They determine the location and size of the distribution of abundances in the fingerprint. Both the Bernoulli and Geometric model fingerprints without the reactivity parameters have a significantly larger deviation than the models with reactivity parameters to the Monte Carlo fingerprints, except for the instances with $r_{A}=r_{B}^{-1}=1.0$. This is to be expected because, in our setup, this corresponds to reactivity parameters of $p_{XY}=1.0$ for all A,B∈{AB}.

Overall, the Geometric model provides a better fit than the Bernoulli model for all fingerprints computed with $r_{A}=r_{B}^{-1}\geq 0.5$: the shapes of the distributions match closely and the deviations to the Monte Carlo fingerprints are the lowest. For fingerprints computed with $r_{A}=r_{B}^{-1}<0.5$, we observe the reverse: the Bernoulli model provides a better fit than the Geometric model for narrow distributions.

The Bernoulli and Geometric models are not only able to compute fingerprints, but also the likelihood of a single copolymer chain. Monte Carlo simulations produce a large random sample of copolymer chains. This allows us to compute and compare the log likelihoods of the sampled data under the different models (Figure 5, right) to further evaluate the models. A model that has a higher likelihood is “closer” to the sample.

Except for $r_{A}=r_{B}^{-1}=1.0$, the log likelihood under the models without reactivity parameters are in all cases lower than their counterparts using reactivity parameters. This is consistent with the fingerprint comparisons. However, contrary to the fingerprint comparisons, the Geometric model has the best log likelihood for all instances.

**Figure 6:**
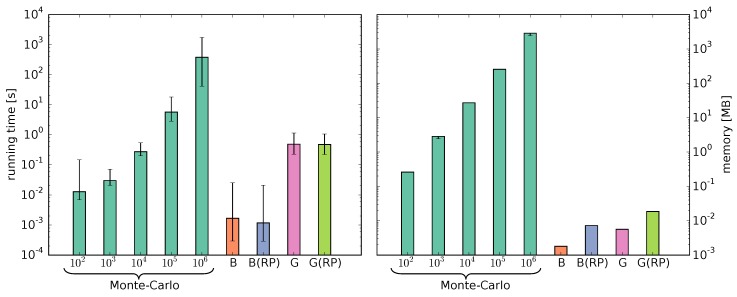
Comparison of the running time (**left**) and memory (**right**) measurements of the Monte Carlo simulations using Gillespie’s algorithm with $10^{2}$ to $10^{6}$ chains and the Bernoulli (B) and Geometric (G) models with and without RP.

The running time and memory requirements of a Monte Carlo simulation increase with the number of simulated chains and for good accuracy the number should be high. The running time and memory of the Bernoulli and Geometric models are determined by the number of synthesis steps. Compared to the theoretical time complexity of $O(T^{3})$ for the Bernoulli model, the Geometric model has a higher theoretical time complexity of $O(T^{5})$. We measured running time (excluding Input/Output operations) and memory of the Monte Carlo simulations with $10^{2}$ to $10^{6}$ chains and of our models (Figure 6).

Computing the Bernoulli model is the fastest. As expected, the measured running time of the Geometric model is higher. However, computing the fingerprints with the Geometric model is still 11.8 and 788.5 times faster than the Monte Carlo simulations with $10^{5}$ and $10^{6}$ chains, respectively. The reactivity parameters have no substantial impact on the running time. Both models require significantly less memory than the Monte Carlo simulations. The additional matrices required for the reactivity parameters increase the memory consumption only slightly.

## 4. Conclusions

We introduced two new Markov chain models: the Bernoulli and Geometric models. The major differences to classical copolymer Markov chains based on the terminal model by Mayo and Lewis [1] are the variable number of added monomers per time step and the time-dependent monomer probabilities. The number of added monomers follows a Bernoulli or geometric distribution, respectively. The reactivity ratio has a major influence on synthesized copolymers and likewise the reactivity parameters of the models play a decisive role.

In our setup, the Geometric model is able to provide a good fit to the fingerprints of the broad polymer distributions, while the fit of the Bernoulli model is particularly good to the mode but not as good to the long tail of the narrow polymer distributions. However, we observe that the likelihood of the copolymer chain sequences is always higher under the Geometric model. This shows that long chains play a major role in characterizing the distribution of copolymer chains.

Our models require less memory than Monte Carlo simulations. The Bernoulli model is faster than Monte Carlo, and the Geometric model is significantly faster (1–3 folds) for a high number of simulated chains, which is necessary for accurate Monte Carlo simulations. In addition, computing our models can be parallelized for multiple cores in a straightforward way, computing different lines of the matrices in parallel.

However, the main advantage of our models over Monte Carlo simulations is that they do not produce just a random sample, but characterize the complete distribution of copolymer chains. Our computations are exact and deterministic. In particular, we can calculate the exact likelihood of any polymer chain. The model parameters can be estimated from measured copolymer fingerprints and compute previously inaccessible statistical properties of the synthesized copolymers. This will be described in a forthcoming publication. Although the Geometric model was more accurate in our setup, the Bernoulli model is a good characterization for copolymer distributions without a long tail and in general can be used as a rapid first estimate.

## Supplementary Materials

Supplementary Materials can be found at www.mdpi.com/2073-4360/8/6/240/s1.

## Abbreviations

The following abbreviations are used in this manuscript:

ODE: Ordinary differential equation
MS: Mass spectrometry
MALDI-TOF MS: Matrix-assisted laser desorption/ionization time-of-flight mass spectrometry
NRMSE: Normalized root mean square error
