# Supplementary material for: New Statistical Models for Copolymerization
Source: Polymers (Basel). 2016 Jun 22;8(6):240. doi: 10.3390/polym8060240 (PMC6432000; doi:10.3390/polym8060240)
Supplement: Supplementary file 1 [file polymers-08-00240-s001.pdf]

Supplementary Materials: New Statistical Models for Copolymerization

Martin S. Engler, Kerstin Scheubert, Ulrich S. Schubert and Sebastian Böcker

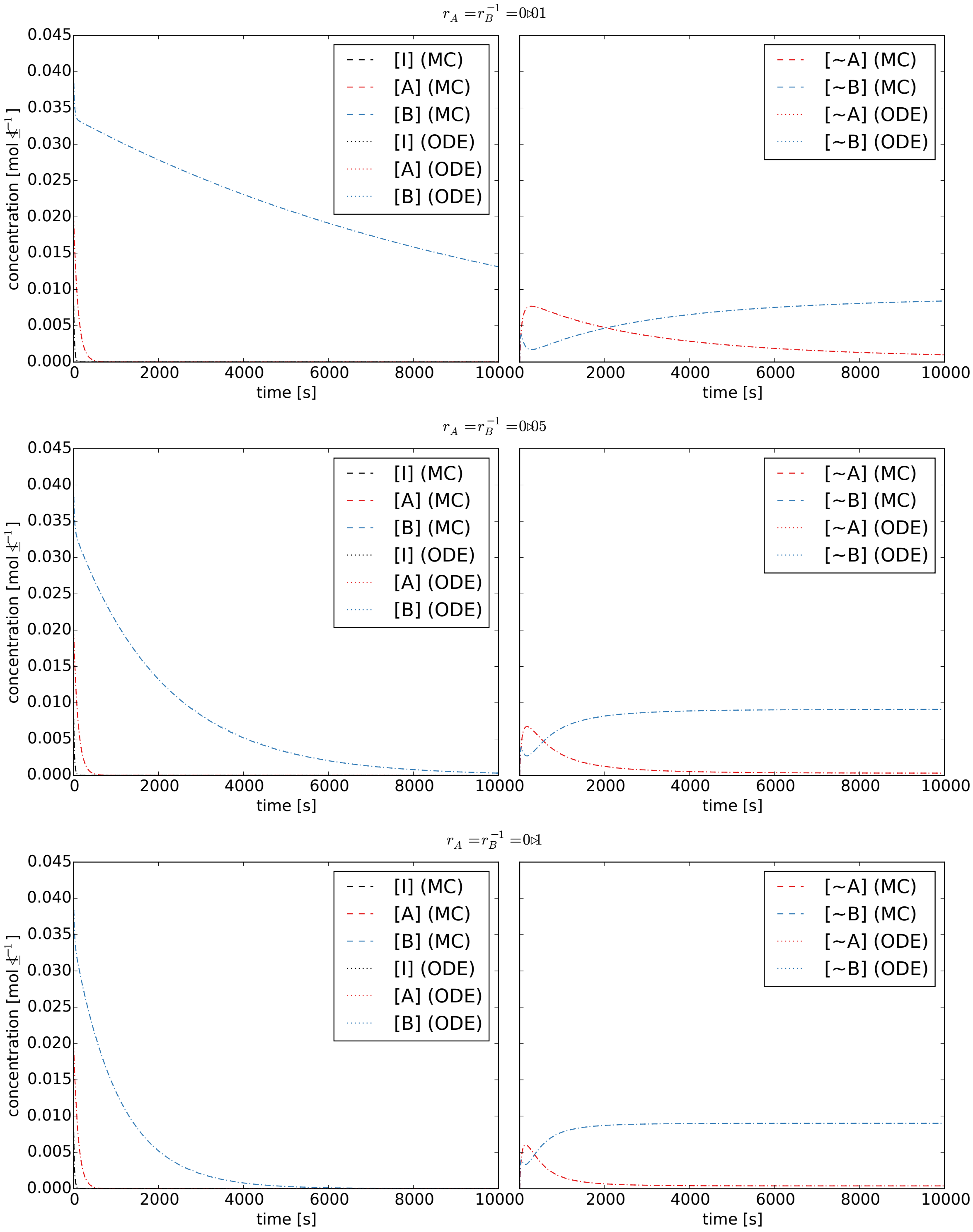

Figure S1. *Cont.*

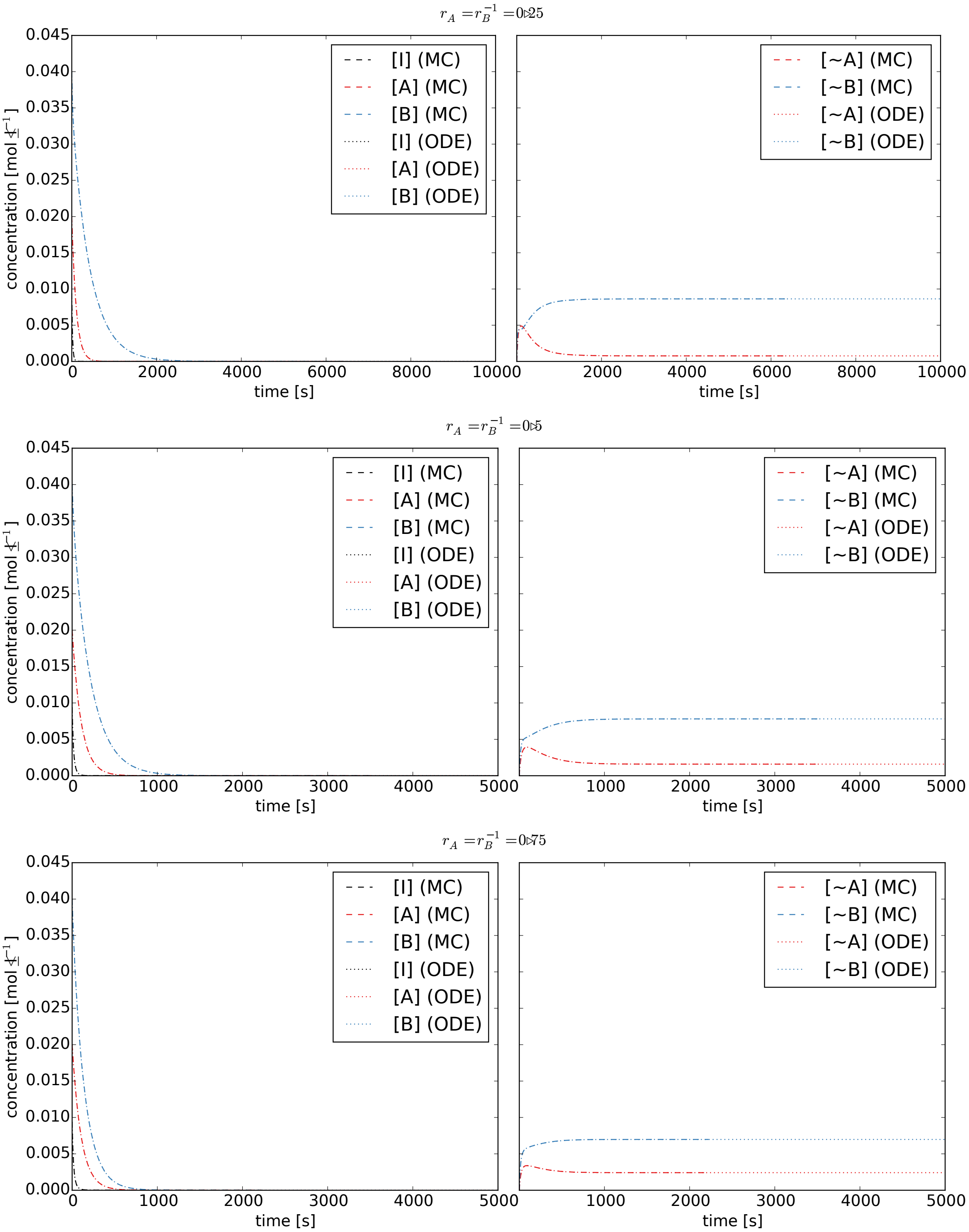

Figure S1. *Cont.*

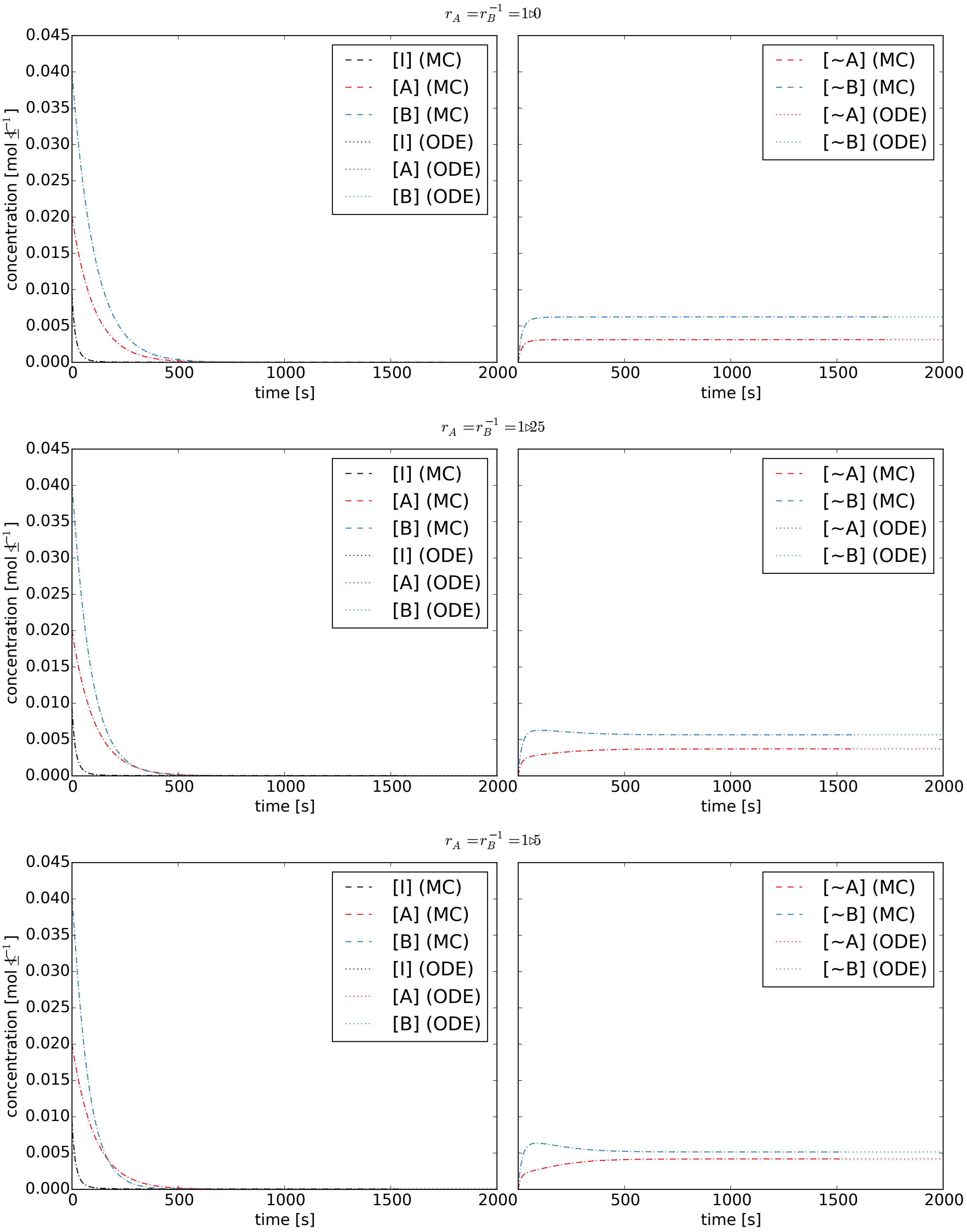

Figure S1. *Cont.*

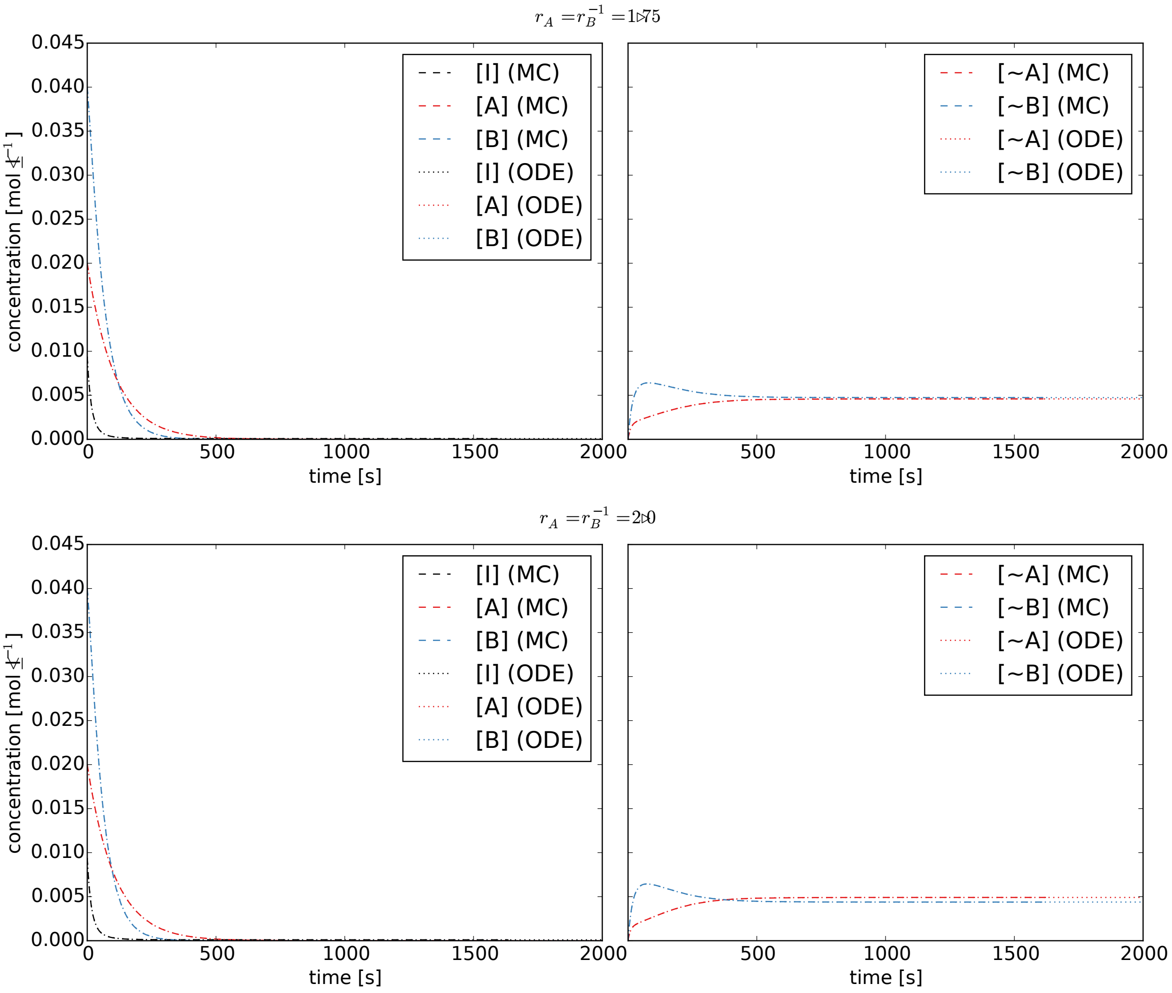

Figure S1. Reactant (**left**) and product (**right**) concentrations simulated by Monte-Carlo (MC) compared to the concentrations computed by solving the ordinary differential equation (ODE) model of the living copolymerization.

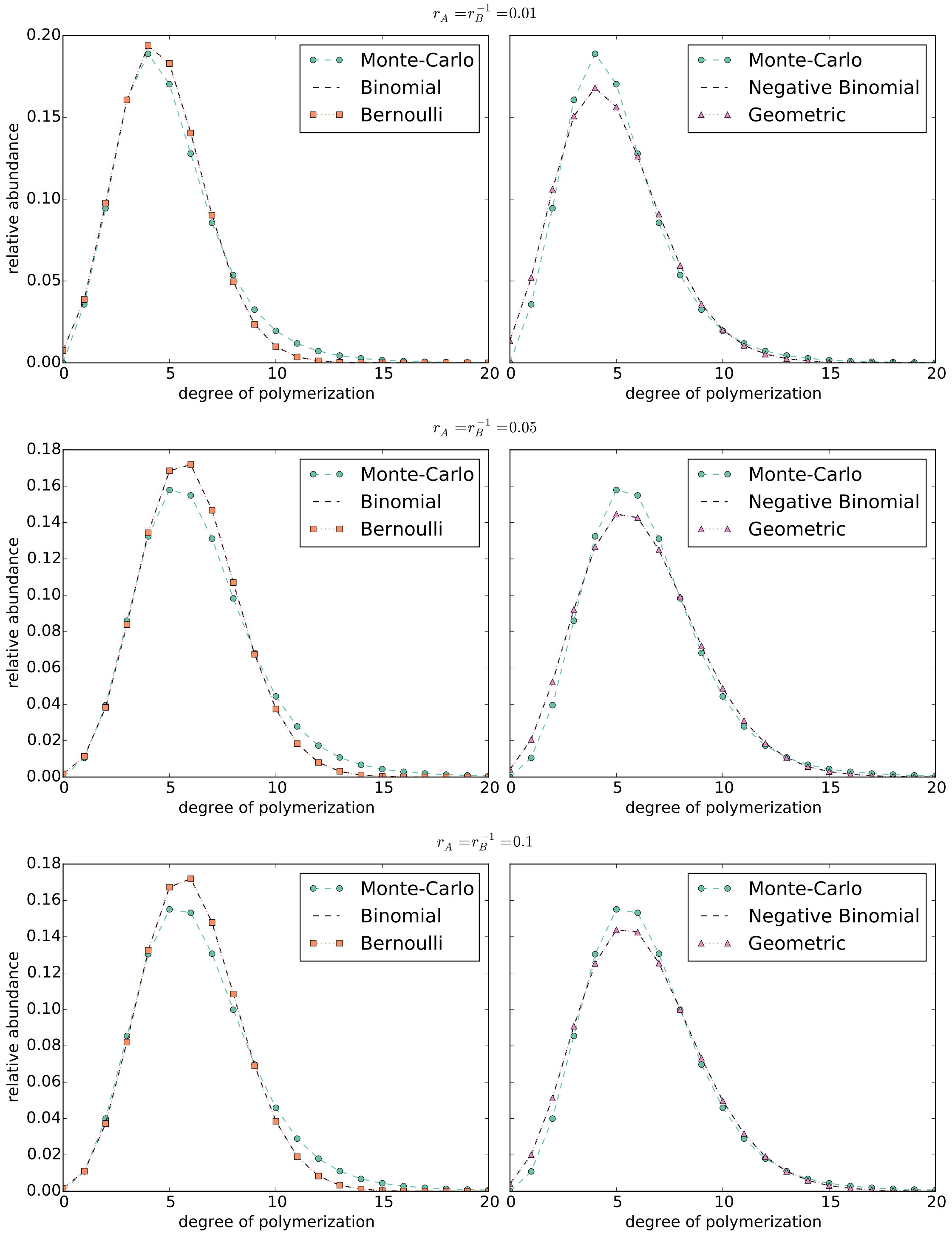

Figure S2. *Cont.*

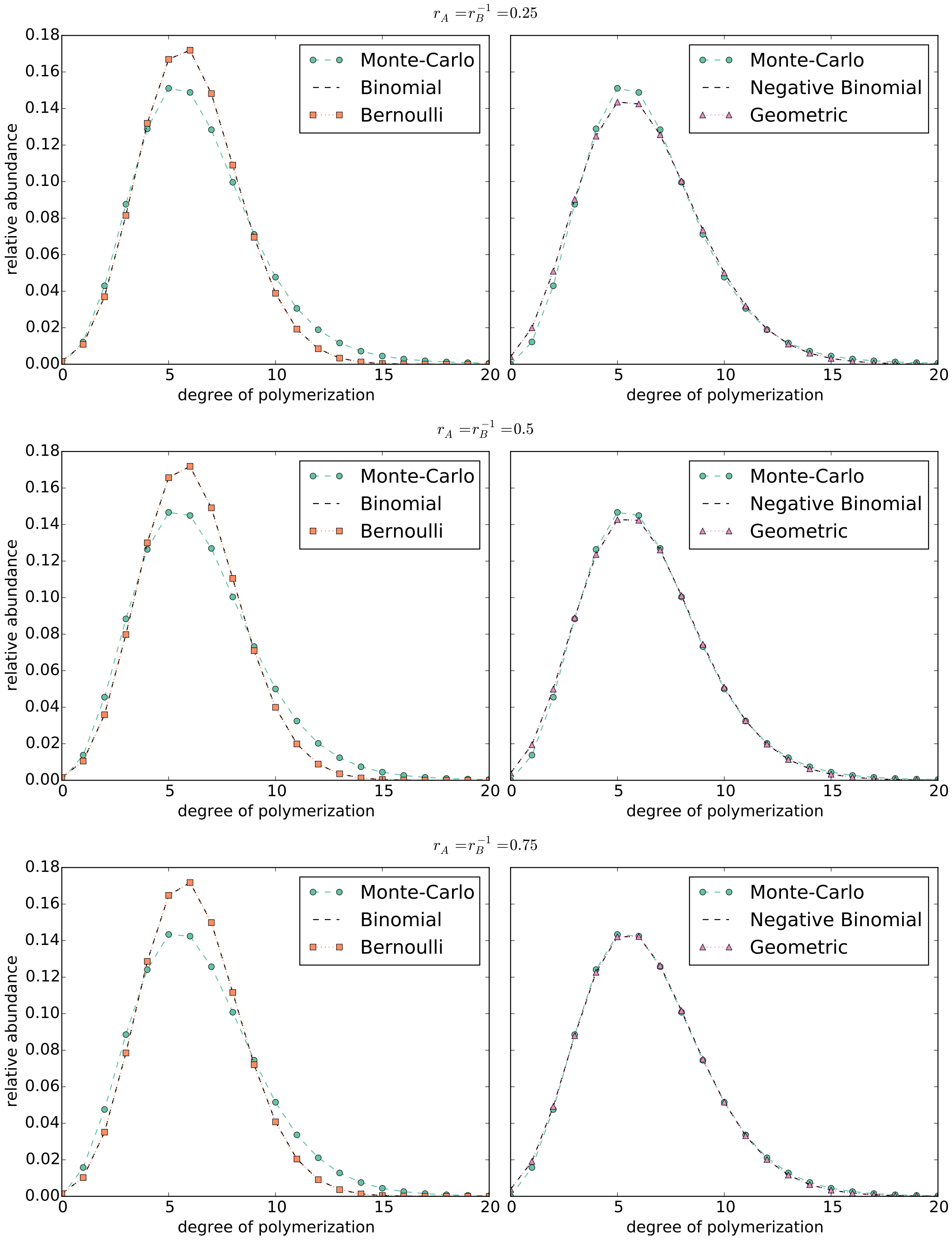

Figure S2. *Cont.*

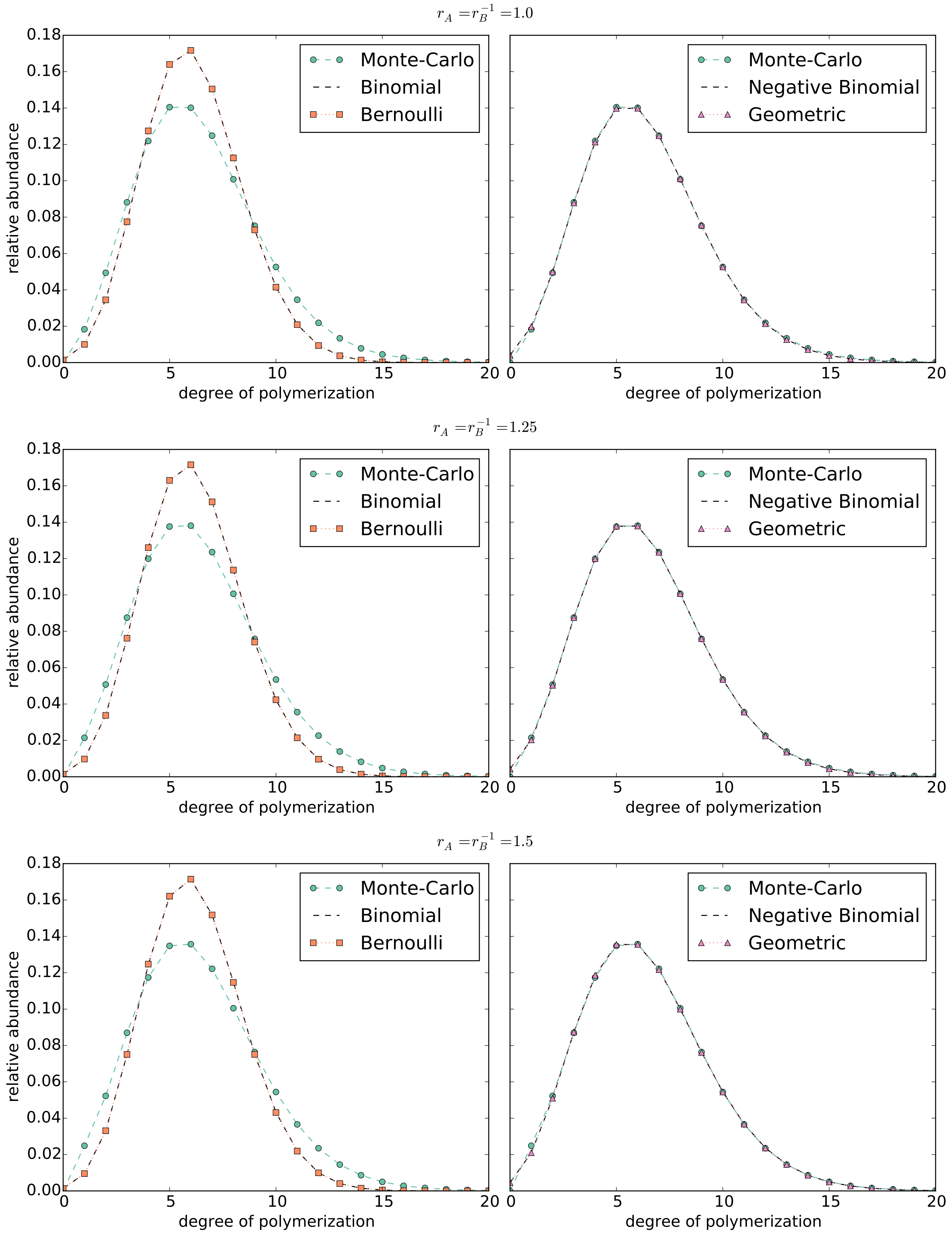

Figure S2. *Cont.*

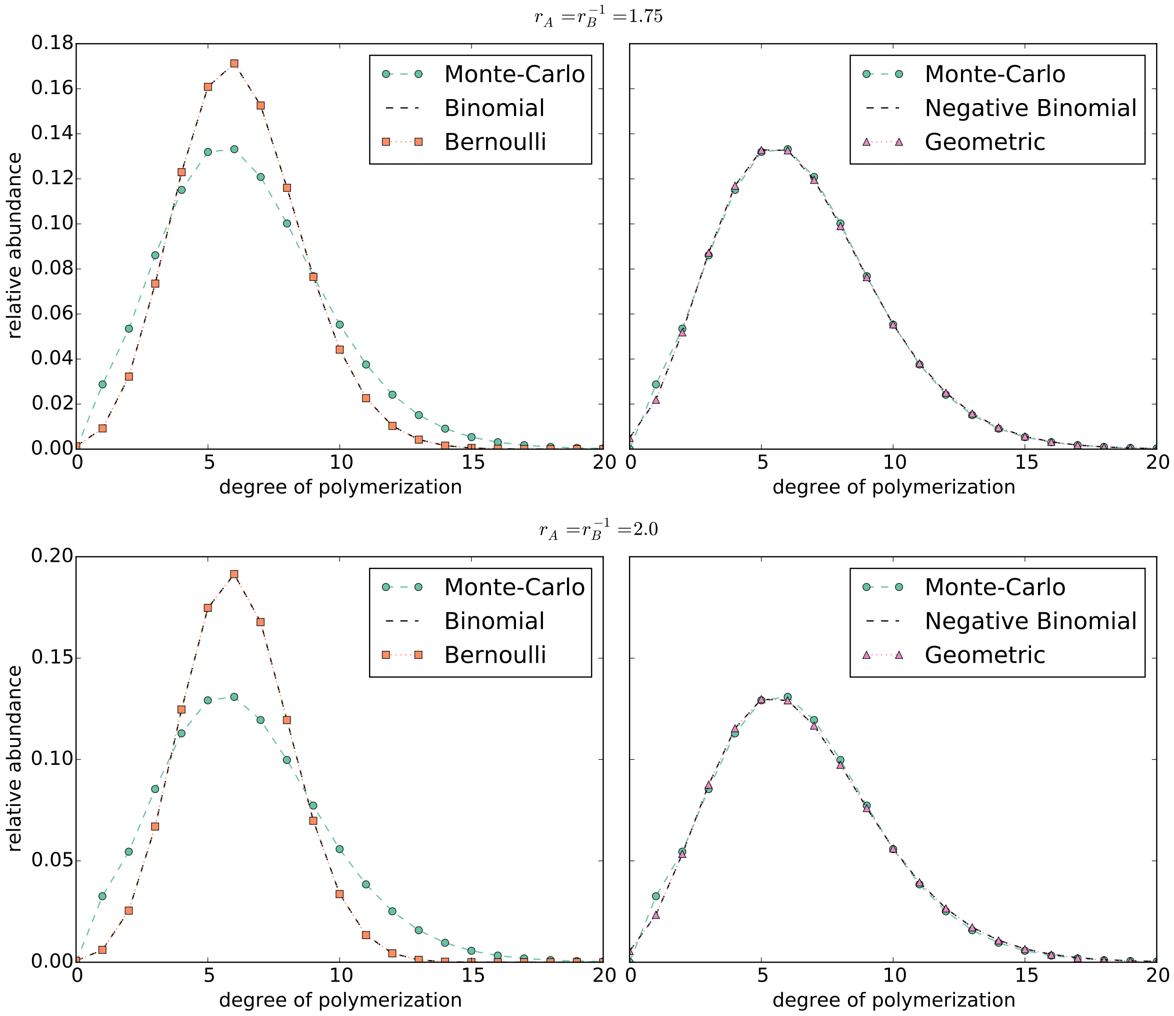

Figure S2. Distribution of chain lengths computed by the Monte-Carlo simulations and the Bernoulli (**left**) and Geometric models (**right**). Additionally, we plotted the binomial and negative binomial probability mass functions to show that the chain lengths computed by the models follow those distributions.

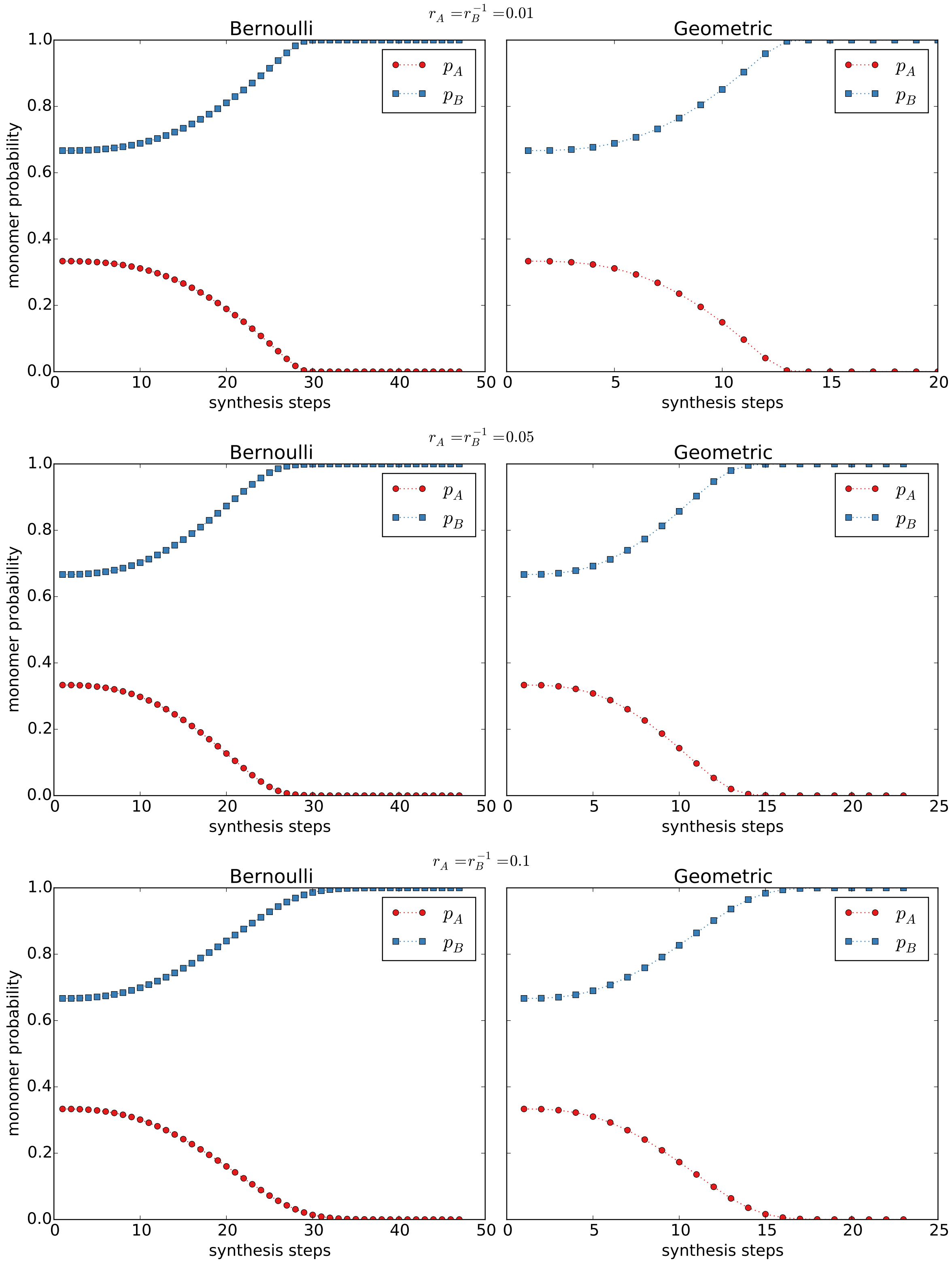

Figure S3. *Cont.*

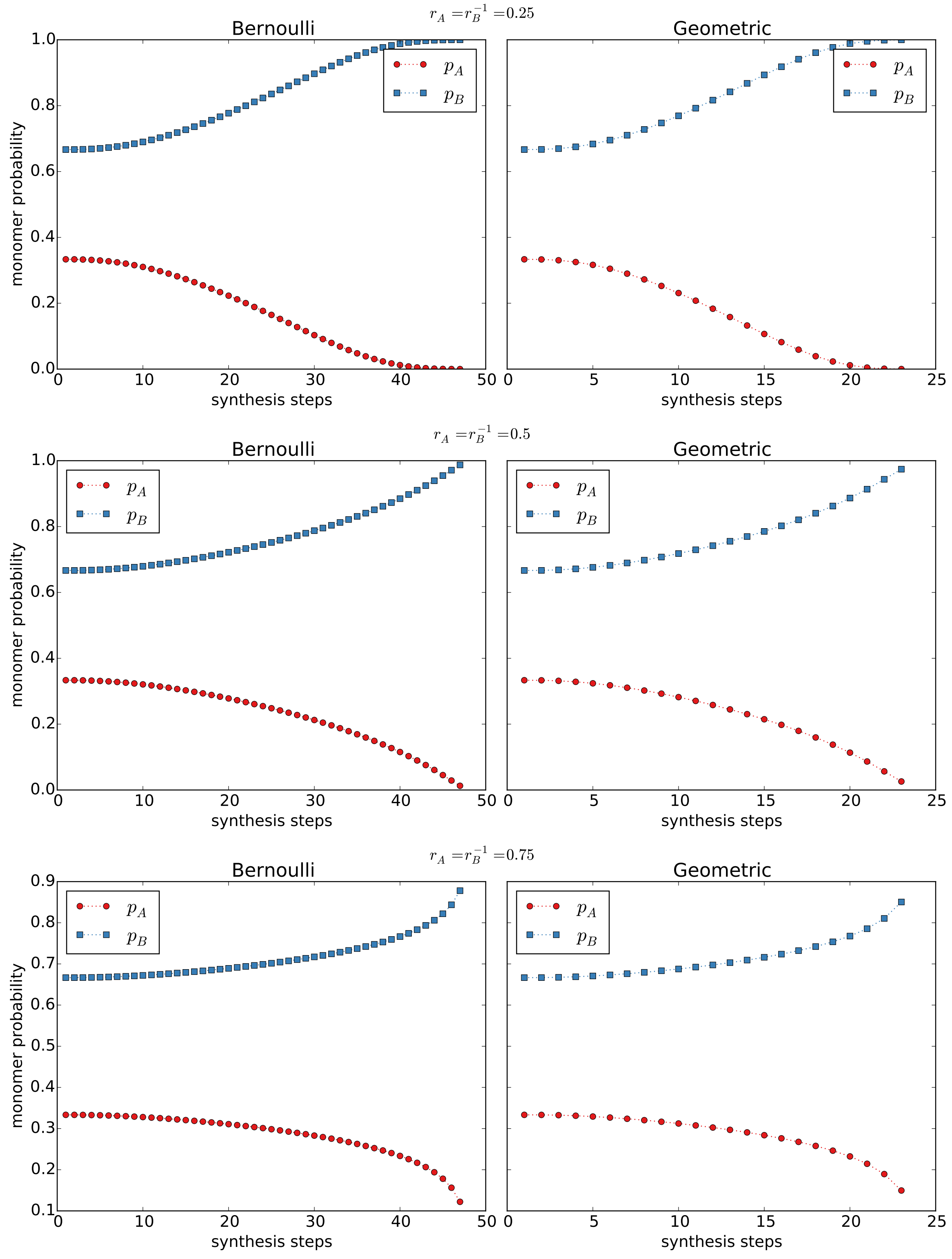

Figure S3. *Cont.*

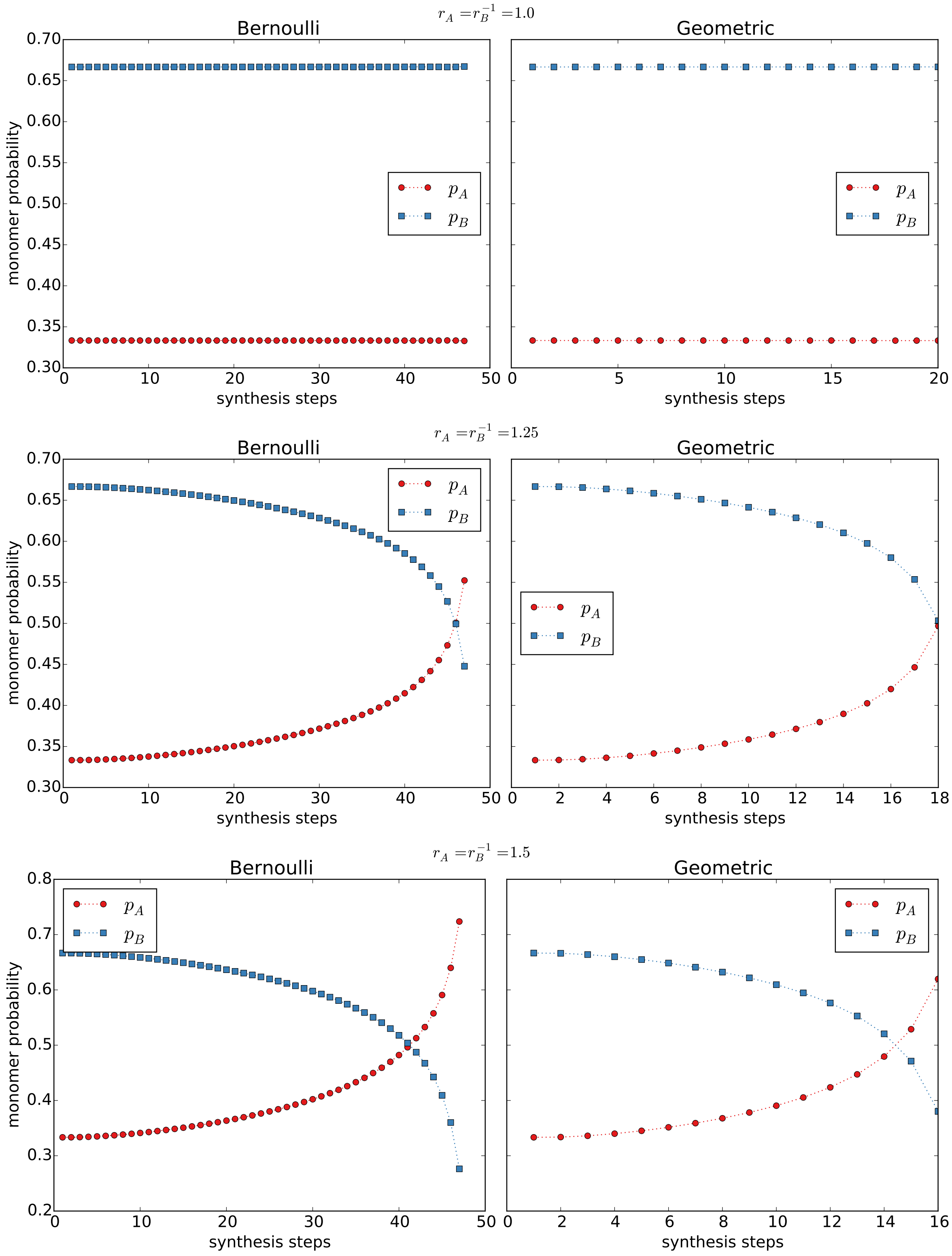

Figure S3. *Cont.*

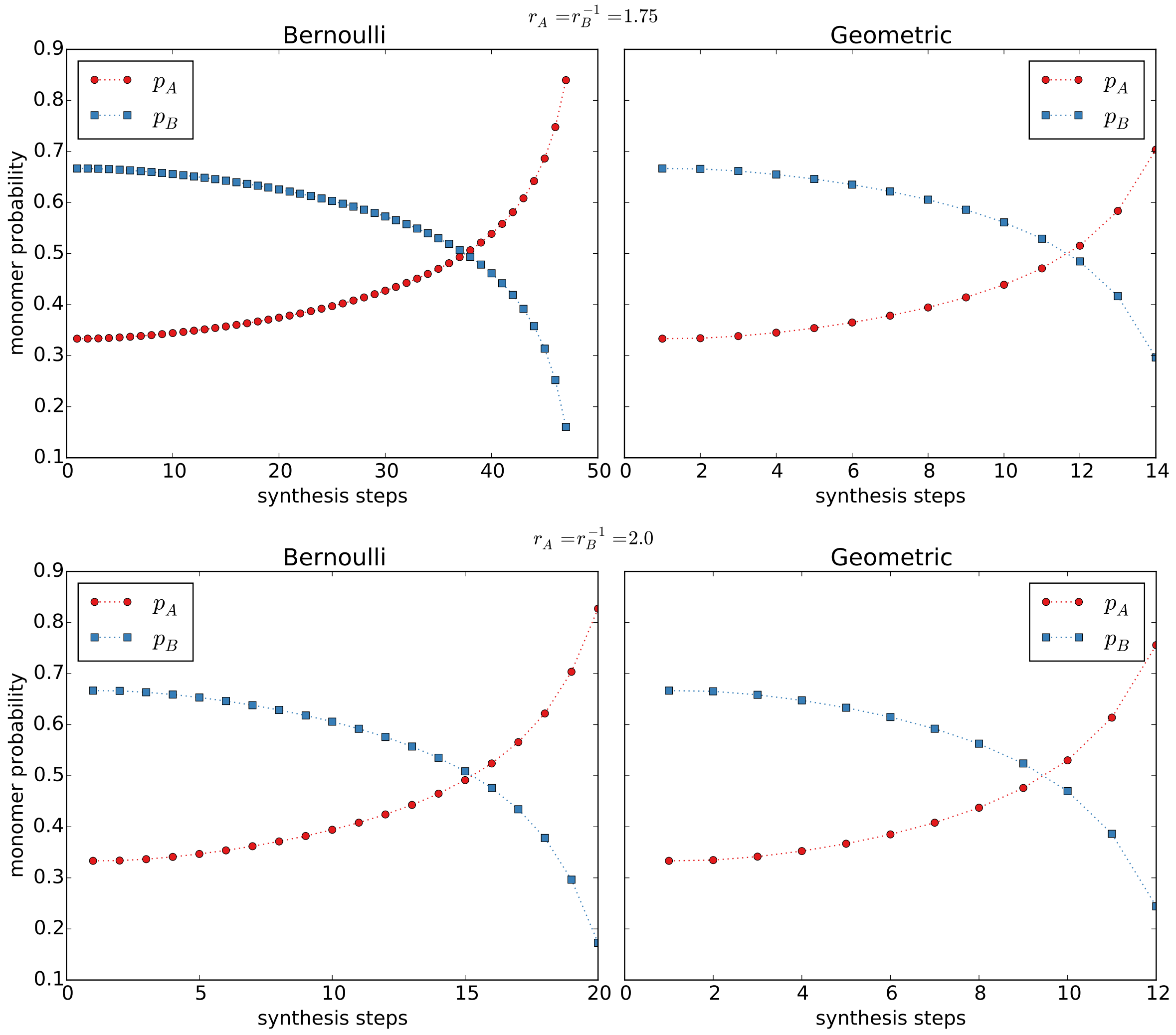

Figure S3. Monomer probabilities p_A and p_B for the Bernoulli (**left**) and Geometric (**right**) models calculated from the average concentrations.

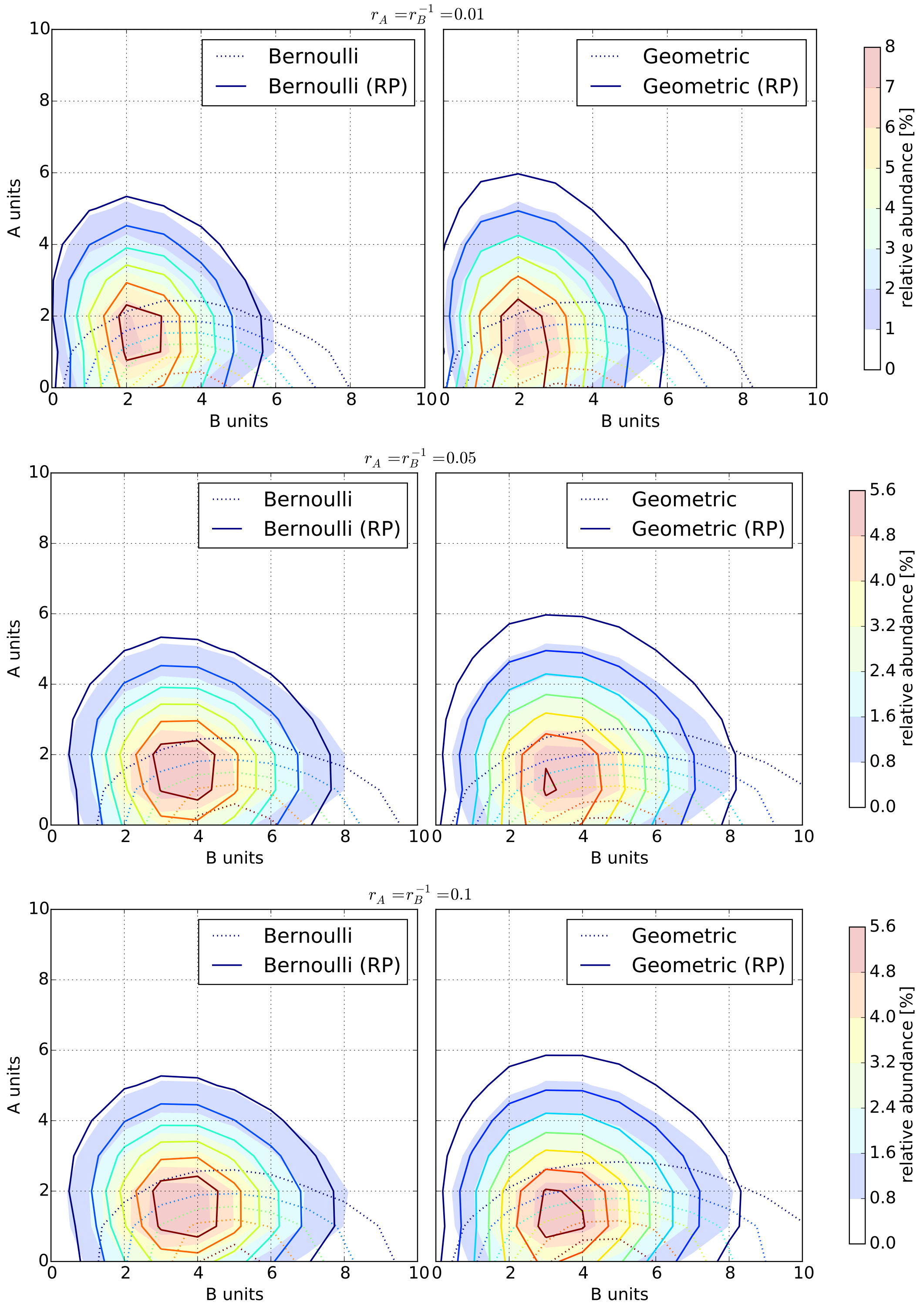

Figure S4. *Cont.*

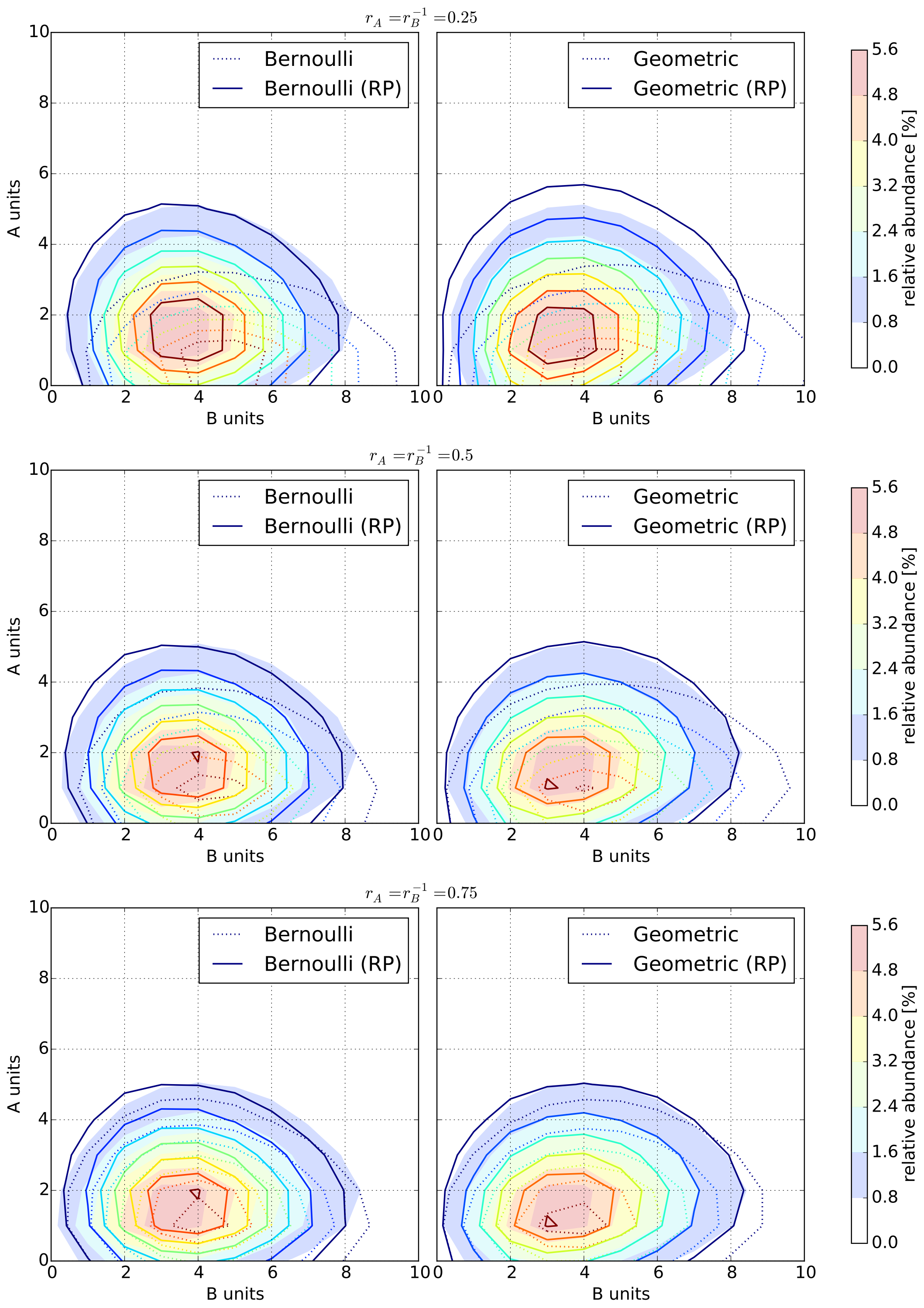

Figure S4. *Cont.*

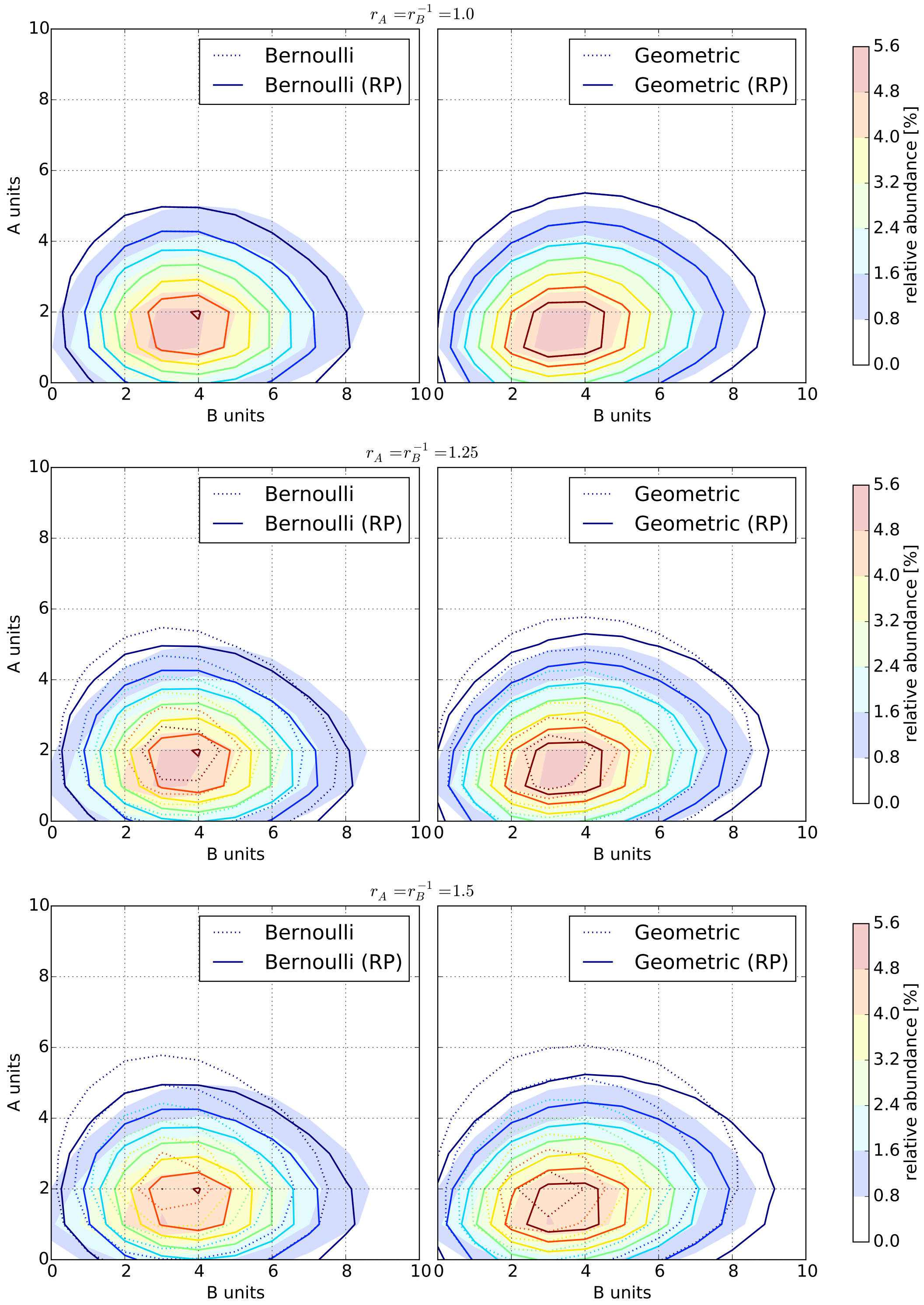

Figure S4. *Cont.*

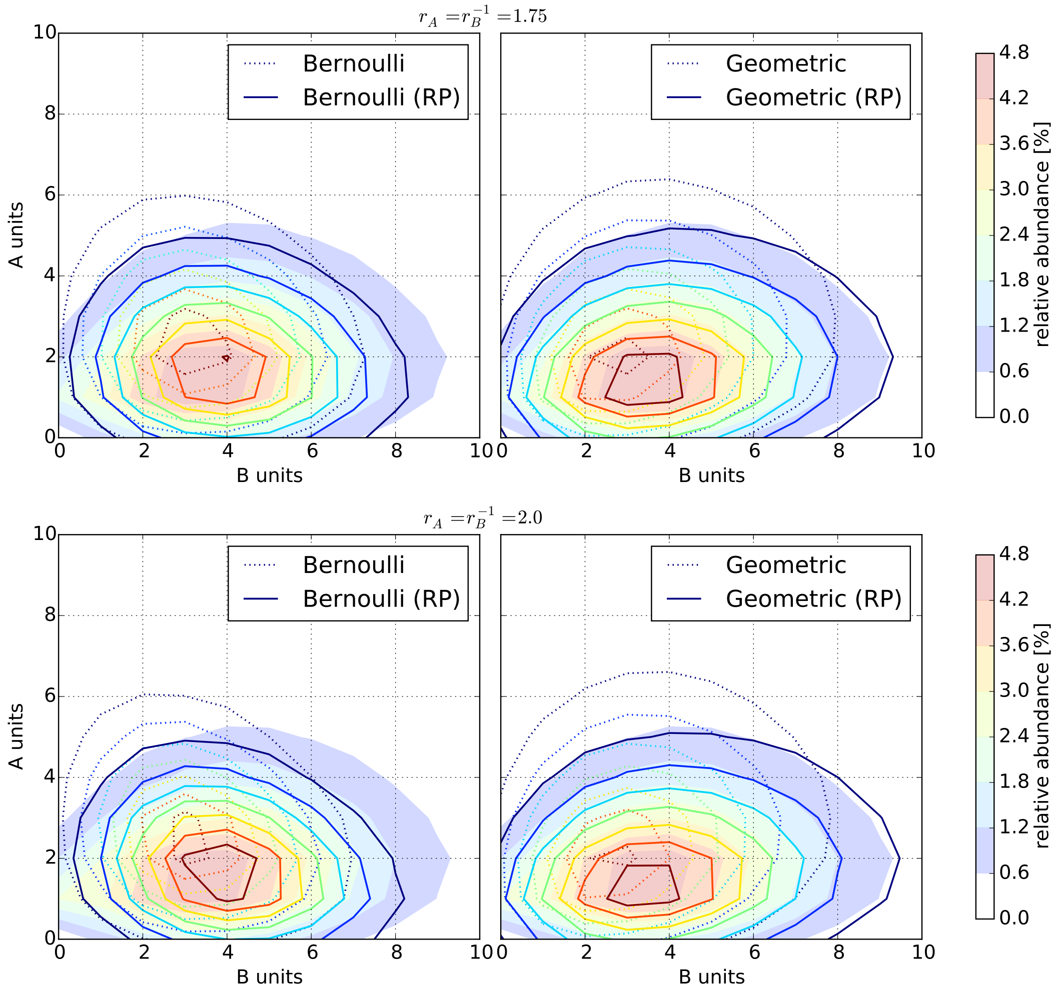

Figure S4. Copolymer fingerprints computed by the Monte-Carlo simulation (filled contours) compared to the fingerprints computed by the statistical models (solid and dashed contours). **Left**: Bernoulli model with and without reactivity parameters (RP); **Right**: Geometric model with and without reactivity parameters (RP).
